# Elevated level of nerve growth factor in the bladder pain syndrome/interstitial cystitis: a meta-analysis

**DOI:** 10.1186/s40064-016-2719-y

**Published:** 2016-07-13

**Authors:** Wei Chen, Da-Yong Ye, Deng-Jun Han, Guang-Qing Fu, Xiang Zeng, Wei Lin, Yong Liang

**Affiliations:** Department of Urology, Zigong No.4 People’s Hospital, Sichuan, China

**Keywords:** Nerve growth factor, Bladder pain syndrome, Interstitial cystitis, Meta-analysis

## Abstract

**Objectives:**

To elucidate the association between nerve growth factor (NGF) level and bladder pain syndrome/interstitial cystitis (BPS/IC) by conducting a meta-analysis.

**Methods:**

We conducted a systematic literature search to identify original studies of NGF level in BPS/IC before November 2015. Eligible studies were retrieved via both computer searches and manual review of references. The summary difference estimates between controlled group and BPS/IC group were calculated based on the weighted mean difference (WMD) with its 95 % confidence interval (CI). Sensitivity and publication analyses were performed after the pooled analysis.

**Results:**

Meta-analysis of 10 original studies involving 295 cases and 290 normal controls showed an increased level of urinary NGF in BPS/IC patients (z = 3.08, P = 0.002). The combined WMD was 36.39 (95 % CI 13.27–59.51). There was significant difference between controlled group and BPS/IC patients in the term of NGF/Cr level (WMD = 0.96, 95 % CI 0.58–1.35; z = 4.89, P < 0.01). There was no significant publication bias in the included studies (P for Begg’s test = 0.73, P for egger’s test = 0.13).

**Conclusions:**

Our results demonstrated that there was an increased level of NGF in the BPS/IC patients.

## Background

Bladder pain syndrome/interstitial cystitis (BPS/IC) is one of the most common chronic disorders of urinary bladder (Bosch and Bosch 2014). The mainly clinical manifestation is urgency and frequency with or without bladder pain. Nowadays, some methods for treatment of this disorder have been applied by doctors, while the long-term effect of these methods remains controversy (Diniz et al. 2013). Early and non-invasive diagnosed method has been put forward in recent years. In these methods, the biomarkers in urinary are the hottest topic discussed. Recent studies showed that nerve growth factor (NGF) is a biomarker which could lead to overactive bladder (OAB) and interstitial cystitis or bladder pain (Liu et al. 2014; Jiang et al. 2013, 2014). However, the results between studies were inconsistent (Seth et al. 2013; Wein 2014; Ochodnicky et al. 2011). In response, we conducted the first meta-analysis by pooling together the results from all published original studies. Our purpose was to examine the diagnostic value of NGF in the BPS/IC.

## Methods

### Literature search

We aimed to identify all publications reporting urinary NGF level in BPS/IC patients. Our literature search was conducted with a systematic literature search before November 2015. The study was in accordance with meta-analysis of observational studies in epidemiology (MOOSE) guidelines (Vandenbroucke 2009). The databases included PubMed, MEDLINE, Springerlink, ScienceDirect, Chinese National Knowledge Internet (CNKI) and EMBASE. The search strategies in our analysis were as following:

#1. (nerve growth factor) OR NGF OR NGFs.

#2. (bladder pain syndrome) OR interstitial cystitis OR BPS OR IC OR BPS/IC OR bladder disease.

#3. #1 and #2.

There was no any language restriction of our literature retrieve, while the trial objective should restrict as human. In order to screening additional relevant data, all the potential related references after paper were manually searched. The manual retrieve procedure was performed by two independent investigators. Any ambiguity among the researchers was resolved by consensus and original data.

### Criteria for inclusion and exclusion

The mainly included criteria for our study were as following: (1) the study should be an original research, which mentioned the urinary NGF level in BPS or IC patients. (2) the primary outcome of NGF level was presented as continuous data, which can be collected to make a pooled quantitative analysis. (3) there was controlled group and corresponding quantitative data in original study. The exclusion criteria were: (a) there was no usable data reported. (b) duplicates data. (c) reviews, narrate or comments. (d) letters to editors, abstracts in conference or non-peer-reviewed journals.

### Data extraction

Two reviewers independently extracted information from each article using standard forms, the extracted data concerned first author, year of publication, location of the study, sample size in controlled group and BPS/IC group, mentioned outcome measures and baseline characteristics. When more than one data were presented in the same study, we separately treated them as different study once the data were no cross effect between different controlled group or different BPS/IC group. Any disagreements were resolved by consensus. The primary outcome was urinary nerve growth factor.

### Quality assessment

The eligibility of each study was assessed independently by two investigators. We assessed the methodological quality of the studies by means of the previously published recommendations for systematic reviews of observational studies. The key points of the current checklist included the following terms: maintenance of comparable groups, baseline of demography, study design, aim of the study, discussion of possible confounders, data collection and bias. Any study that met fewer than four criteria present should be excluded.

### Statistical analysis

All the statistical procedure were performed in STATA software version 12.0 (STATA, College Station, TX, USA). The weighted mean difference (WMD) was used in the evaluation of difference urinary NGF level between controlled group and BPS/IC group. Moreover, the WMD also used in the evaluation of ratio of NGF and serum creatinine (Cr). The 95 % confidence interval (CI) of WMD was calculated to identify the study outcome variation. It is considered statistically significant for outcome measure when the 95 % CI excluded zero. Heterogeneity was assessed by the Cochran’s Q test, with a 10 % significance level. On the other hand, the *I*^2^ statistic was adopted to interpret the degree of heterogeneity. A fixed effect model was performed in the data comparison section unless the heterogeneity degree was *I*^2^ > 50 %, in which case a random effects approach was used to adjust for the combined estimate. Potential publication bias was assessed by Egger’s line regression and Begg’s funnel plot. Sensitivity analysis was performed by excluding the older studies. For all meta analyses, two side P < 0.05 was considered statistically significant.

## Results

**Fig. 1:**
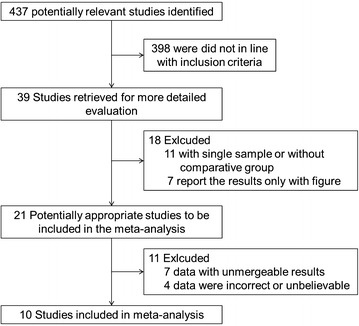
Study selected flow diagram

A total of 437 articles were retrieved initially, there were 398 papers were did not in line with our inclusion criteria, 11 relative papers were exclusion after read the titles and abstracts. 7 papers of remained 28 studies report the results only with figure, 7 papers report the data with unmergeable results, and data in the other 4 studies were incorrect or unbelievable due to their primary designs. Finally, we identified a total of ten studies (Baykara et al. 2003; Liu and Kuo 2007; Liu et al. 2009, 2010; Boudes et al. 2011; Chung et al. 2011; Liu and Kuo 2012; Tyagi et al. 2012; Jiang et al. 2013, 2014) mentioned urinary NGF level in BPS/IC patients according to our above mentioned criteria. A detail flow diagram of our search and selection was shown in Fig. 1. Characteristics and quality assessment of these studies are summarized in Table 1.

**Table 1:** Basic characteristics of included studies

Author	Year	Region	Sample size		Outcome	Study design	Quality
			BPS/IC	Control			
Hsin-Tzu Liu	2012	Taiwan	30	28	Urinary NGF, NGF/Cr	Compared	6
Pradeep Tyagi	2012	USA	10	10	Urinary NGF	Compared	5
Mehmet Baykara	2003	Turkey	9	7	Urinary NGF	Compared	5
Hsin-Tzu Liu	2007	Taiwan	19	19	Urinary NGF	Compared	6
Hsin-Tzu Liu	2009	Taiwan	58	28	Urinary NGF, NGF/Cr	Compared	5
Chia-Yen Chen	2010	Taiwan	28	28	Urinary NGF, NGF/Cr	Compared	6
Chia-Yen Chen	2010	Taiwan	28	28	Urinary NGF, NGF/Cr	Compared	6
Shiu-Dong Chung	2011	Taiwan	22	33	Urinary NGF, NGF/Cr	Compared	5
Shiu-Dong Chung	2011	Taiwan	22	33	Urinary NGF, NGF/Cr	Compared	5
Mathieu Boudes	2011	Belgium	6	5	Urinary NGF	Compared	5
Yuan-Hong Jiang	2013	Taiwan	30	26	Urinary NGF	Compared	5
Yuan-Hong Jiang	2014	Taiwan	33	45	Urinary NGF, NGF/Cr	Compared	5

*NGF* nerve growth factor, *NGF/Cr* ratio of nerve growth factor and creatinin

295 cases and 290 controlled participants were involving in our study. The number of original BPS/IC patients was ranged from 6 to 58, and the number of controlled participants was range from 5 to 33. There was no gender, age or body mass index (BMI) dividing in 10 studies. Thus our study cannot make subgroup analysis based on these demographic characteristics. Of all the included studies, seven studies (Liu and Kuo 2007; Liu et al. 2009, 2010; Chung et al. 2011; Liu and Kuo 2012; Jiang et al. 2014) were conducted in Taiwan, China. One (Tyagi et al. 2012) study was from USA, one study (Baykara et al. 2003) from Turkey and the other one was in Belgium (Boudes et al. 2011). The range of publication year was range from 2003 to 2014.

**Fig. 2:**
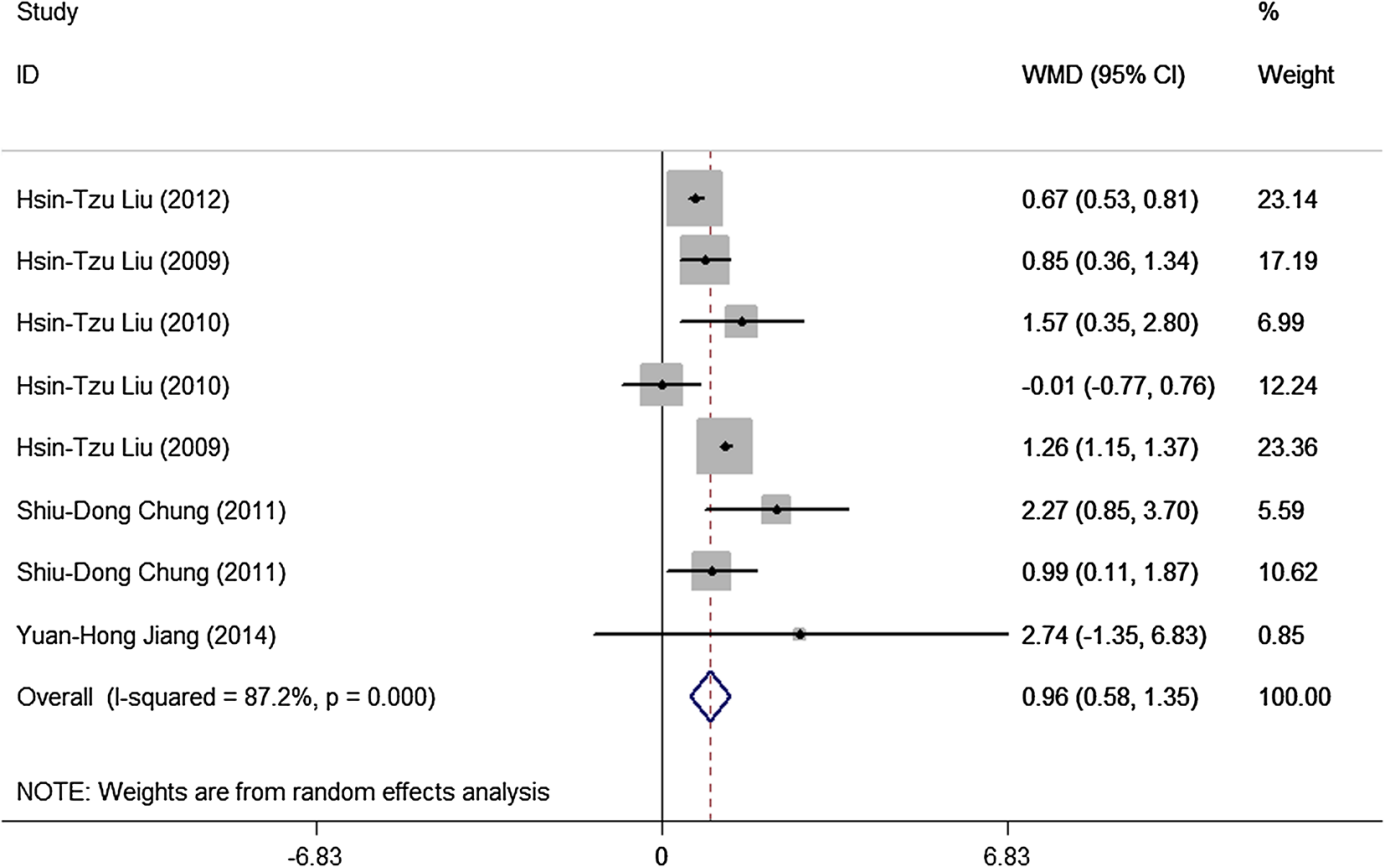
Meta-analysis of urinary NGF in the diagnosis of BPS/IC. The size of the square is proportional to the percent weight of each study in the meta-analysis; the *horizontal lines* represent 95 % CI

**Fig. 3:**
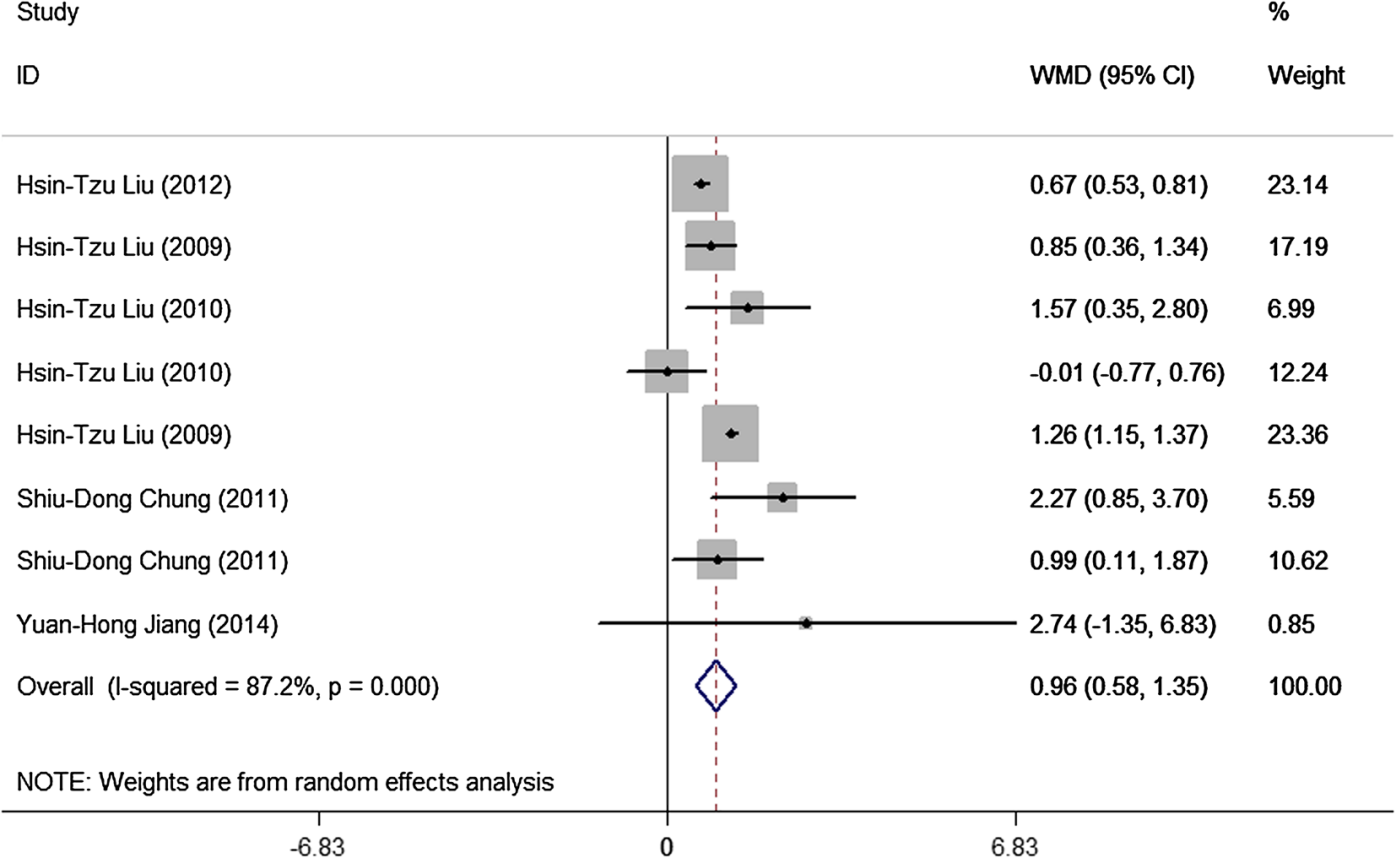
Meta-analysis of urinary NGF/Cr in the diagnosis of BPS/IC. The size of the square is proportional to the percent weight of each study in the meta-analysis; the *horizontal lines* represent 95 % CI

Ten studies reported the urinary NGF level in BPS or IC patients. In the selected studies, BPS or IC patients seemed to have a high urinary NGF level when compared with controlled participants (WMD = 36.39, 95 % CI 13.27–59.51; z = 3.08, P = 0.002), Fig. 2 showed the difference of urinary NGF level in BPS/IC group and controlled group. Eight studies mentioned the ratio of urinary NGF and Cr. In the selected studies, the level of NGF/Cr was significant higher in BPS/IC patients when compared with controlled participants (WMD = 0.96, 95 % CI 0.58–1.35; z = 4.89, P < 0.01), Fig. 3 showed the difference of NGF/Cr level in BPS/IC group and controlled group.

We also conducted a sensitivity analysis to investigate the robustness of our findings. In our analysis, we excluded the oldest study published by Mehmet Baykara in 2003. The result showed that the difference between groups was as same as previous (WMD = 39.80, 95 % CI 15.13–64.48; z = 3.16, P = 0.002).

**Fig. 4:**
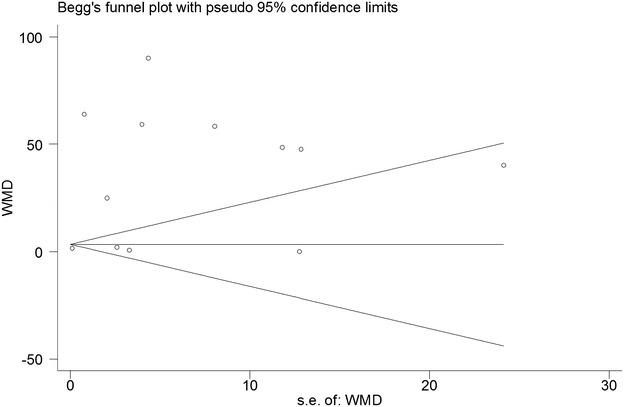
Begger plot for the assessment of potential publication bias for urinary NGF in the diagnosis of BPS/IC

We were able to assess publication bias in the primary outcome. The degree of asymmetry was not statistically significant by both Begg’s test and Egger’s line regression (P for Begg’s test = 0.73, P for egger’s test = 0.13). The Begg’s funnel plot was shown in Fig. 4.

## Discussion

It is well-known that the non-invasive diagnostic method of urinary tract disorder is rapidly development in recent years. The most common method is the urinary biomarker in the diagnosis (Rubio-Diaz et al. 2009). There are various biomarkers which included nerve growth factor and nuclear matrix protein. The level of NGF in bladder pain and interstitial cystitis were inconsistent among previous studies.

We have pooled all available related evidence to discuss the diagnostic value of NGF in BPS/IC through the meta-analysis method (Seth et al. 2013; Liu et al. 2009). In this analysis, we identified that serum NGF level and NGF/Cr level in BPS/IC patient were significantly higher than that in controlled participants.

Recent investigations into the pathophysiology of BPS/IC have demonstrated elevated levels of several bladder and urinary biomarkers in this bladder disorder, such as nerve growth factor (NGF) (Ochodnicky et al. 2011; Chung et al. 2011). Urinary NGF is produced from the urothelium and bladder smooth muscles. Patients with idiopathic detrusor overactivity, neurogenic bladder or inflammatory bladder diseases such as BPS/IC have been reported to have increased bladder sensation and urinary NGF levels (Jiang et al. 2013). NGF is responsible for the growth and maintenance of sensory neurons and appears to play a role in neuroimmune interactions, in tissue inflammation, and in neuroplasticity for neuronal events leading to OAB (Chung et al. 2011).

To the best of our knowledge, there is no a comprehensive assessment of the relation between NGF levels and the incidence of BPS/IC. This is the first meta-analysis of original studies on the potential relationship between NGF levels and the incidence of BPS/IC. Here, we pooled ten studies involving 585 participants to get a more stable and creditable result. The results suggested there was an increased level of NGF in the BPS/IC patients. To be noted, no substantial heterogeneity was detected in those studies included in our present analysis. On the basis of Egger’s and Begg’s tests, we have shown an absence of publication bias in these meta-analyses. In addition, sensitivity analyses showed none of the studies considerably affected the summary associations between NGF levels and the incidence of BPS/IC.

Data are liable to be confounded by many factors, which could be reflected by the heterogeneity among studies. Firstly, our meta-analyses were based on the overall category of IC/BPS, we cannot distinguishing the subtypes based on the original study. Secondly, the publication year between studies were significant different. We know that the detection rates of NGF were different in different year because of the difference of awareness and assay technique of these diseases, even though with a same diagnostic standard. Thirdly, most of the studies are coming from the same study group (Liu HT, Jiang YH and Chung SD). While we have checked that the patients in the different studies were not the same, and the data in different studies were independent. Fourth, study with small sample size also can lead to this heterogeneity. The heterogeneity was inevitably significant when merging such a confidence interval. Finally, our study relies on nonrandomized and retrospective data. These reasons also could lead to potential bias of our result.

In summary, our meta-analysis of all relevant original studies showed a significant increase level of NGF in BPS/IC patients. While the further studies are also required to better confirm the findings.

## Abbreviations

CI: confidence intervals
FEM: fixed effect model
REM: random effects model
WMD: weight mean difference
BPS/IC: bladder pain syndrome/interstitial cystitis
OAB: overactive bladder
NGF: nerve growth factor
CNKI: Chinese National Knowledge Internet
Cr: serum creatinine
